# Guided growth for correction of knee flexion deformity: a series of four cases

**DOI:** 10.1007/s11751-011-0110-7

**Published:** 2011-07-22

**Authors:** B. A. MacWilliams, B. Harjinder, P. M. Stevens

**Affiliations:** 1 Motion Analysis Laboratory, Shriners Hospitals for Children-Salt Lake City, Fairfax Rd. @ Virginia St., Salt Lake City, UT 84103 USA; 2 Department of Orthopaedic Surgery, University of Utah, Salt Lake City, UT USA; 3 Primary Children’s Medical Center, Salt Lake City, UT USA

**Keywords:** Fixed knee flexion deformity, Crouch gait, Guided growth, Hemiepiphysiodesis, 8-plate

## Abstract

Fixed knee flexion deformity can present as an insidious and significant problem in diverse etiologies, most commonly in cerebral palsy. Traditional surgical intervention has included posterior capsulotomy and supracondylar femoral osteotomy, both of which carry significant associated morbidity and risks. In the skeletally immature patient, guided growth may be used to correct or substantially diminish the deformity. We are presenting our early experience encompassing four subjects who completed instrumented gait analysis both prior to and after distal femoral anterior guided growth (hemiepiphysiodesis). Changes in gait and function resulting from surgery in each individual are reported. Outcomes indicate improved knee range of motion and alleviation of crouch at the knee with secondary improvements in the ankle, hip and pelvis. Three subjects with initially slow gait velocity improved to within normal limits by demonstrating increased stride length. A measure of overall gait kinematics showed improvements in all limbs. Anterior guided growth (hemiepiphysiodesis) of the distal femur resulted in positive quantitative changes in all four patients, though degree and types of changes were variable in this small series. Encouraged by these findings, we now prefer guided growth to extension supracondylar osteotomy for the skeletally immature patient with fixed knee flexion deformity.

## Introduction

Fixed knee flexion deformity (FKFD) can present as an insidious and significant problem in diverse etiologies like CP, arthrogryposis and myelomeningocele, with CP being relatively more common [1–3]. Conservative management including physical therapy and bracing, often combined with Baclofen or selective and temporary chemical denervation of muscles using botulinum toxin or phenol may be prescribed to address more mild presentations [4, 5]. However, the natural progression of the impairment is from muscle spasticity to contracture and eventually bony deformity [6]. Co-spasticity of muscles acting across joints and the development of musculoskeletal deformities make gait more laborious and energy consuming [7].

Surgical options after failure of conservative management include multi-level soft tissue and bony procedures, bipolar frame distraction, supracondylar osteotomy, anterior distal femoral hemiepiphysiodesis or guided growth [6, 8–12]. The effect of supracondylar extension osteotomy is neutralized at a rate of about 1° per month by rapid growth; recurrence has also been reported following frame distraction [13, 14].

The benefits of gradual correction of FKFD by guided growth using the flexible construct of the 8-plate has been documented in a previous article by the senior author (PMS) [15]. This article focuses on gait analysis data that include physical examination for a comprehensive assessment of outcome. This is the first report that utilizes gait analysis to objectively assess correction of FKFD after anterior distal femoral guided growth.

## Materials and methods

**Fig. 1:**
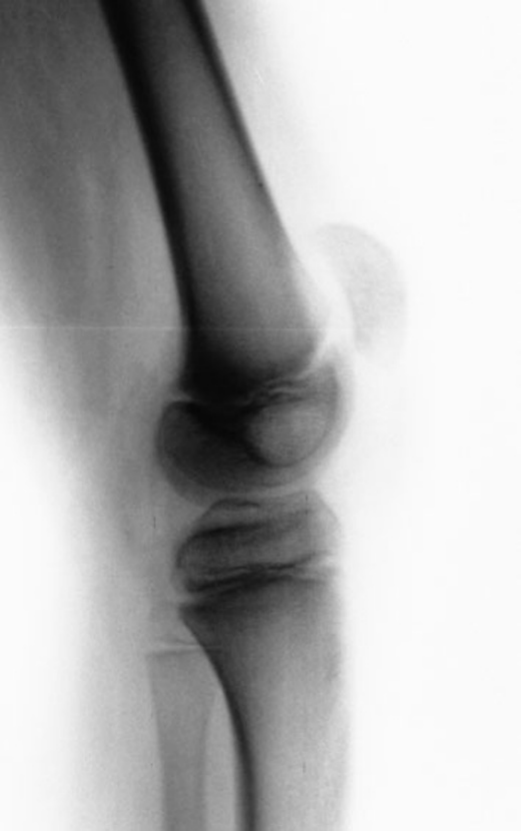
Bilateral fixed knee flexion deformity of 18° causing obligatory crouch gait and associated anterior knee pain (subject 1)

Four patients, all men, with a diagnosis of FKFD secondary to an underlying neurological or congenital disorder participated in this prospective IRB-approved study. Subject demographics are given in Table 1, and a preoperative radiograph of subject 1 is provided in Fig. 1. A standard marker model was applied, and data were collected using a ten camera motion capture system (Vicon, Centennial CO, USA) and four force platforms (AMTI, Watertown, MA, USA) as the subjects ambulated at a self-selected velocity along a 10-m walkway [16]. A standard physical examination by an experienced therapist was performed to measure passive range of motion at the knee, hip and ankle. The Gross Motor Function Measure (GMFM) Questionnaire sections D and E (standing/balance and walking) [17, 18] and the Gillette Functional Assessment Questionnaire (FAQ) [19] were administered to families, and the patients’ Gross Motor Function Classification System [20] was rated by a physical therapist. Within 1 month following the preoperative gait study, each subject subsequently underwent anterior distal femoral guided growth, placing a plate on either side of the patello-femoral sulcus.

**Table 1:** Subject demographics

Subject	Diagnosis	Side(s)	Age at pre	Implant time (months)
1	Diplegia	Bilateral	11 (5)	24
2	Arthrogryposis	Bilateral	12 (9)	12
3	Diplegia	Bilateral	14 (2)	13
4	CDP	Left	9 (5)	29

Age is given in years (months)

*CDP* congenital dislocated patellae

### Technique

**Fig. 2:**
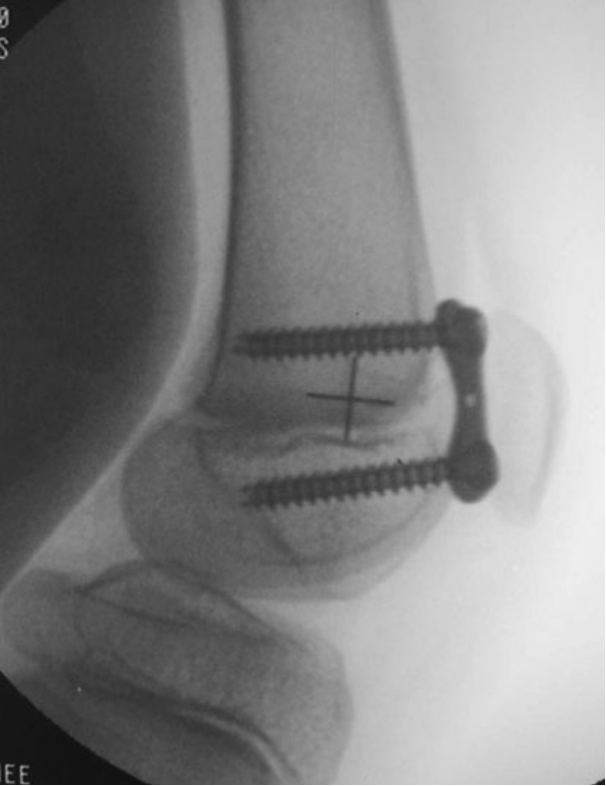
Intraoperative fluoroscopic view demonstrating the positioning of the anterior 8-plate (subject 1), one is placed on each side of the patello-femoral sulcus. They are intracapsular but non-articular

**Fig. 3:**
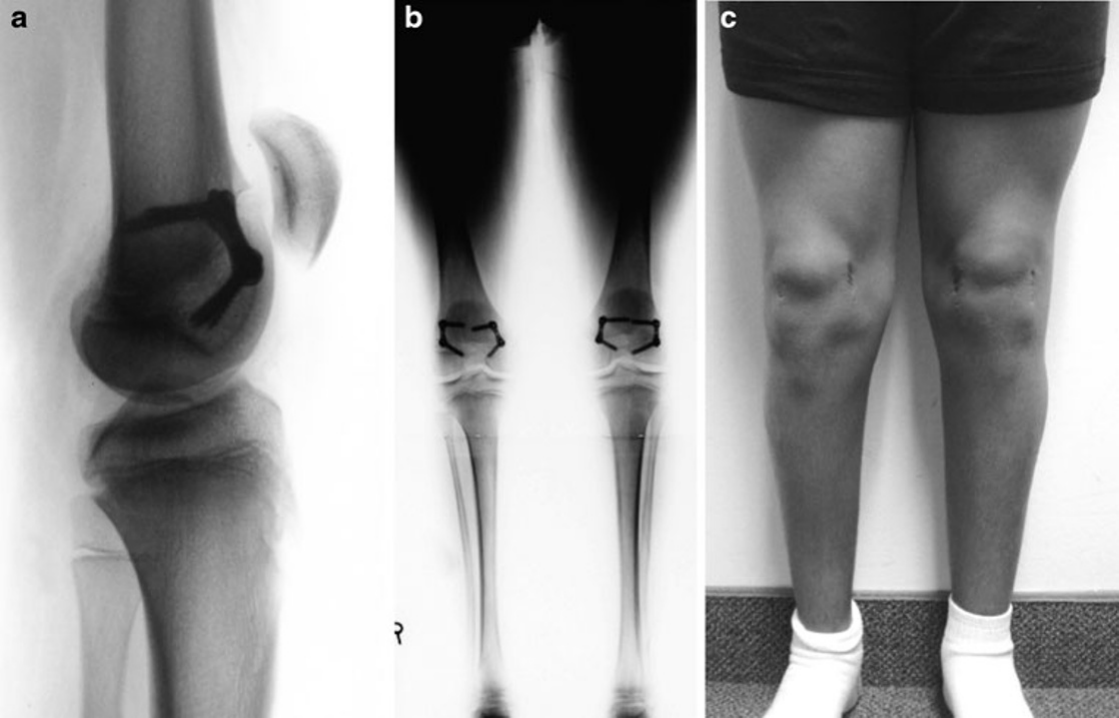
**a** Lateral view documenting full correction of the fixed knee flexion deformity. Despite the residual patella alta, he has no pain, patellar instability, or related functional limitations; therefore, the patellar tendon does not need to be advanced. **b** A full-length radiograph showing the retained 8-plates. **c** Clinical appearance at the time of follow-up gait analysis. Upon full correction of the fixed knee flexion deformity, the 8-plates are typically removed. All images are from subject 1

Under tourniquet control, using fluoroscopic guidance, the distal femoral physis was identified; this is best observed in the lateral, cross-table projection. Two incisions about 3 cm long were made on either side of the patella, centered over the physis. The capsule and synovium were opened, the femoral sulcus visualized, and the plates placed just outside the articular portion of the joint surface. The central hole of the 8-plate was slipped over the Keith needle, which was inserted into the physis. Two 1.6-mm guide wires were then passed through the proximal and distal holes of the plate under fluoroscopic guidance, taking care not to violate the physis or the joint. The cortex was drilled (5 mm), and self-tapping, cannulated, 24-mm screws were inserted. Upon removal of the guide pins, the screws were further tightened to countersink them within the plate. The screws need not be parallel, and the implants were intracapsular, but not on the articular surface (Fig. 2). The wound was closed, soft dressing applied, and the patient was allowed ambulation as tolerated. Follow-up films were taken at 4 weeks to assure the position and integrity of the plates (Fig. 3). The patients were followed at 3–6 month intervals. When full passive extension was achieved, the gait analysis study and physical examination were repeated. Within 1 month following the gait study, plates were removed (average plate insertion time 15.07 months, range 11–23 months).

### Data analysis

Due to the small number of participants in this study, data are presented individually. Gait analysis measures focus on key sagittal plane parameters during stance. Additionally, an overall measure of gait kinematics is reported using the gait deviation index [21]. Two physical examination measures of the knee flexion contracture and popliteal angle are also provided. Using all treated limbs, means and confidence intervals are presented for the following kinematic variables: knee and hip flexion at initial contact, maximum knee and hip extension in stance, and GDI. Gait data from a typically developing group of similar aged (8–12 year old) children (*n* = 38) collected from a separate IRB-approved study are given for comparison.

## Results

**Fig. 4:**
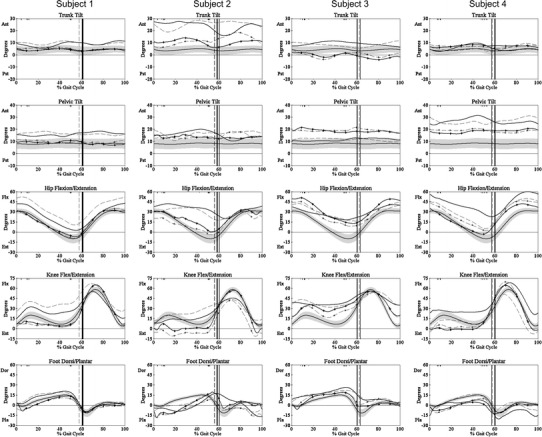
Sagittal kinematics during gait of each subject. Each line represents an average of five trials. Preoperative data have no *symbol*, and postoperative data are starred. *Left side* data are solid, and *right side* data are dashed. *Gray solid line* and *shaded band* indicate means and one standard deviation from a pediatric control group

**Fig. 5:**
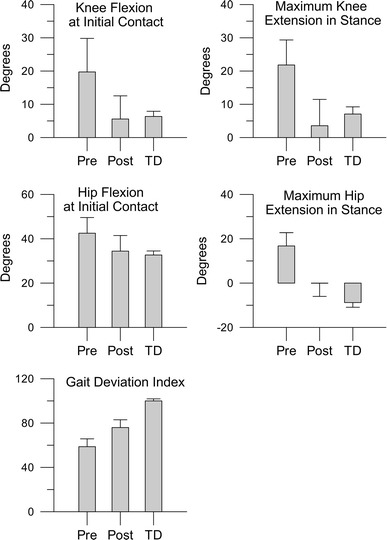
Means of selected key kinematic variables to assess crouch gait profile and GDI for the seven limbs studied. *Error bars* represent the 95% confidence interval. Typically developing (TD) data are given to refer the changes from surgery

In this study, varying degrees of improvement in both the fixed deformities as measured during physical examination and by sagittal knee kinematics during gait were determined. Degree of knee flexion contracture and popliteal angle from physical examination are reported in Table 2; velocity, cadence and stride length are reported in Table 3. Complete sagittal plane gait data are presented in Fig. 4 with statistical evaluation of group results of key measures presented in Fig. 5. Results are summarized first individually then by group.

**Table 2:** Physical examination results and gait deviation index (GDI)

Subject	Side	Knee flexion		Popliteal angle		GDI	
		Pre	Post	Pre	Post	Pre	Post
1	L	7.5	7.5	−35	−40	79.8	101.9
	R	10	7.5	−30	−40	68.7	101.3
2	L	15	0	−45	−45	48.6	65.2
	R	15	0	−40	−45	47.4	50.5
3	L	22.5	10	−60	−50	56.2	65.9
	R	20	0	−65	−45	62.2	74.7
4	L	27.5	0	−35	−30	48.0	72.1
Ave		16.8	3.6*	−44.3	−42.1	58.7	75.9*

GDI is a normalized score determined from nine kinematic variables where values >90 are within one standard deviation of an age-matched control set, and each 10 points represents differences of one standard deviation

Popliteal angle is measured from the vertical reference

*Significantly different changes from pre- to post-analysis

**Table 3:** Temporal–spatial gait parameters and functional tests

Subject	Velocity (m/s)		Cadence (steps/s)		Stride length (m)		GMFCS		GMFM-66		FAQ	
	Pre	Post	Pre	Post	Pre	Post	Pre	Post	Pre	Post	Pre	Post
1	1.25	1.04	1.06	0.92	1.18	1.13	1	1	100	100	10	10
2	0.86	1.13	1.09	1.10	0.79	1.02	3	2	63	67	9	9
3	0.63	1.19	1.01	1.02	0.62	1.17	1	1	85	78	8	9
4	0.97	1.23	1.01	1.01	0.96	1.22	1	1	100	100	10	10

Velocity is measured in meters per second, cadence in steps per second and stride length in meters

*GMFCS* Gross Motor Function Classification System, *GMFM-66* 66-item normalized Gross Motor Function Measure sections D and E, *FAQ* Gillette Functional Activity Questionnaire

### Subject 1

In subject 1, an individual with diplegic CP, no change in the left side and only a slight improvement in fixed knee flexion deformity on the right side were observed by physical examination; yet, this subject experienced a complete correction according to his gait curves. His GDI indicated that his gait returned to within normal control values, an improvement in more than two standard deviations. He did not improve temporally or functionally, but was normal in these measures prior to surgery. Thus, in this case, the underlying neurological deficit was mild, and a slight correction of the bony deformity enabled a normal walking pattern.

### Subject 2

In subject 2, an individual with arthrogryposis, large changes in fixed knee deformity (15° bilaterally) were documented, and his gait pattern was changed from crouch gait to back-kneeing. This subject made significant gains in velocity, and improved his GMFCS from a level 3 to a level 2 ambulator as he was able to walk without use of a crutch after surgery, though GMFM-66 and FAQ scores were stable. In this case, correction of the FKFD may have unmasked underlying quadriceps weakness, resulting in the recurvatum. This can, however, be controlled by orthoses and is considered a good outcome. Physical examination revealed that ankle range of motion did not change, remaining at a maximal dorsiflexion of neutral with the knee extended or flexed. Post-surgical kinetics revealed reduced ankle moment support, indicating that recurvatum did not result from an increased ankle plantarflexion–knee extension couple. Radiographs indicate vertical tali, which may contribute to persistent abnormal ankle kinematics.

### Subject 3

In subject 3, while FKFD improved markedly by physical examination (12.5° on the left and 20° on the right), these improvements translated into more minor gains in crouch gait compared with subject 1. Overall gait kinematics improved by one standard deviation, considering a clinically significant gain [22], and were most obviously realized in improved pelvis and trunk positions. The subject had large temporal improvements, as velocity nearly doubled and returned to within normal limits with gains resulting from improvement in stride length. This subject’s function decreased after surgery as GMFM-66 decreased by seven points due to a poorer performance in standing and balance (85%) in contrast to walking (94%). In particular, this subject decreased in ability to balance on a single leg and rise from the floor. This might be attributed to this subject’s autism and ADHD and related pharmaceutical treatment. Despite the loss in balance, walking ability according to his FAQ score improved from 8–9. Additionally, at a later evaluation (4 years following surgery), his GMFM-66 score increased to 92, indicating a gain from his preoperative study. This subject also complained of significant anterior knee pain prior to surgery, which was resolved.

### Subject 4

Subject 4 was the only unilateral subject, having bilateral congenital dislocated patellae, but knee flexion contracture and epiphysiodesis of the left limb only. This subject’s knee contracture improved from 27.5° to 0° and resulted in alleviation of his crouch gait pattern, though a straight leg stance pattern resulted. Like subject 2, there was no kinetic evidence of an ankle plantarflexion–knee extension couple. This subject’s velocity and stride length improved to within normal limits, and GDI improved more than two standard deviations.

Comparisons of key variables on the seven limbs studied revealed significant alleviation of the crouch gait imposed by FKFD.

### Physical examination

Knee flexion contractures improved in all but one limb, and as a group, changes were statistically significant (Table 2, *P* = 0.027). Popliteal angles improved in the subject with the greatest degree of contracture (subject 3), but were otherwise variable, and not statistically significant (*P* = 0.63). It is noteworthy that this technique does not produce the loss of knee flexion that is typically seen after posterior release of supracondylar osteotomy. In fact, the arc of motion is effectively increased.

### Temporospatial and standardized functional measures

Temporal measures improved to within normal limits (1.26 ± 0.28 m/s) with surgery in subjects 2, 3 and 4 with increases in velocity resulting from increases in stride length (Table 3). Velocity in subject 1 decreased, but as a result of reduced cadence, it was within normal limits at both evaluations. GMFCS level improved in subject 2 from a level 3 to level 2, but decreased in subject 3 from a level 1 to level 2.

### Kinematics

Sagittal knee curves were completely normalized in subject 1 (Fig. 4). Secondary to this improvement, trunk, pelvis, hip and ankle also improved to within normal limits. In subjects 2 and 4, crouch was alleviated but transformed to a recurvatum knee pattern. In subject 3, gains in knee extension at initial contact were apparent, but less dramatic throughout stance phase. Marked improvements were noted throughout stance at the trunk, pelvis and ankle. The gait deviation index improved in all sides of all patients, with values of subject 1 improving to within control limits (>90).

Analysis of the seven limbs indicated that both knee flexion at initial contact and maximum knee extension in stance values both improved reflecting the relief of crouch gait (Fig. 5). At the hip, extension at initial contact decreased indicating less compensation necessary to achieve adequate step length. Maximal hip extension improved reflecting relief of crouch gait. In all limbs, each of these variables that were initially outside the normal range (±1 standard deviation) showed improvement. Those variables that were initially within normal range remained within normal range.

## Discussion

Knee flexion contractures significantly alter the kinematics of walking and dramatically reduce the comfort and efficiency of ambulation. Co-spasticity of muscles acting across joints and the development of musculoskeletal deformities make gait more laborious and energy consuming [23]. This also increases the load on the hip and knee joints. Progressive flexion places more force on the quadriceps, leading to overstretching of the muscle fibers and the infrapatellar tendon, causing patella alta, patellar fragmentation, chondromalacia, joint instability, muscle weakness in terminal extension and pain secondary to patellofemoral degenerative joint disease [24, 25]. The benefits of guided growth for FKFD have been recently documented [15].

This minimally invasive technique is well tolerated by an already-compromised population. Our current preference is to offer distal femoral guided growth to address the fixed knee deformity. We no longer favor the aggressive approach of distal femoral osteotomy/patellar tendon advancement in the growing patient. Because these patients have experienced excellent relief of knee pain and improved gait, we have accepted patella alta as a radiographic finding without clinical significance once full knee extension has been restored. Likewise, we do not recommend concomitant hamstring recession, deferring this decision until the plates are removed. Parents and physical therapists are counseled preoperatively that, although the incisions are small and immediate ambulation is permitted, children may experience some pain and crepitance around the anterior plates that are just beneath the extensor retinaculum. These symptoms generally abate within 6–12 weeks. If the deformity recurs after hardware removal, the procedure is easily repeated.

This is but a small sample of patients at our institutions who have undergone distal femoral guided growth to correct fixed knee flexion deformity. Gait analysis is not routinely ordered for these individuals as their primary orthopedic problem is obvious. This report focuses on just four patients who did undergo both pre- and postoperative gait analysis.

This report focuses on six key variables that most directly measure the effects of anterior guided growth for knee contraction. The physical examination measure of knee flexion contracture by goniometer is presented along with five variables from instrumented gait analysis. Each of the gait variables that were initially abnormal improved in each patient with surgery. Knee flexion contractures measured by a physical therapist improved in all but one subject limb. It is likely that the initial measures in this subject were inaccurate as radiographically 18° of contracture was initially determined, and the surgeon determined 20–25° of contracture bilaterally. This subject also showed marked improvements in gait parameters following surgery. A residual knee contracture due to hamstring tightness may have been present postoperatively and is supported by worsening in popliteal angles recorded. Dynamic loading during gait may create moments about the ankle and knee sufficient to overcome this latent contracture as measured during passive range of motion examination.

In this preliminary study of outcomes of four patients, improvements were found in all patients following guided growth to correct fixed knee flexion deformity. Improved range of motion at the knee markedly improved stride length and self-selected velocity, and alleviation of the crouch gait pattern is reported. Along with improvement in knee kinematics during ambulation, secondary improvements at the hip, pelvis and trunk are also noted. Increases in velocity and stride length occurred in the three subjects (2, 3 and 4) who had presented with less than normal values preoperatively. Though specific changes are somewhat diverse, all four cases are viewed as good or excellent outcomes.
